# Pediatric post COVID-19 condition: an umbrella review of the most common symptoms and associated factors

**DOI:** 10.1093/eurpub/ckae033

**Published:** 2024-02-26

**Authors:** Aurora Heidar Alizadeh, Mario Cesare Nurchis, Jacopo Garlasco, Alessandro Mara, Domenico Pascucci, Gianfranco Damiani, Maria Michela Gianino

**Affiliations:** Department of Health Sciences and Public Health, Section of Hygiene, Università Cattolica del Sacro Cuore, Roma, Italy; Department of Woman and Child Health and Public Health, Fondazione Policlinico Universitario A. Gemelli IRCCS, Roma, Italy; Department of Public Health and Paediatrics, Università di Torino, Torino, Italy; Department of Public Health and Paediatrics, Università di Torino, Torino, Italy; Department of Health Sciences and Public Health, Section of Hygiene, Università Cattolica del Sacro Cuore, Roma, Italy; Department of Woman and Child Health and Public Health, Fondazione Policlinico Universitario A. Gemelli IRCCS, Roma, Italy; Department of Health Sciences and Public Health, Section of Hygiene, Università Cattolica del Sacro Cuore, Roma, Italy; Department of Woman and Child Health and Public Health, Fondazione Policlinico Universitario A. Gemelli IRCCS, Roma, Italy; Department of Public Health and Paediatrics, Università di Torino, Torino, Italy

## Abstract

**Background:**

Although the long-term consequences of the Coronavirus Disease-2019 (COVID-19) pandemic are yet to be fully comprehended, a syndrome symptomatically akin to the COVID-19 disease has been defined, for children and adolescents, in February 2023 by the World Health Organization (WHO) as ‘post COVID-19 condition’ (PCC). Potential consequences of COVID-19 that affect developmental milestones in children and adolescents should be comprehended in their magnitude and duration. The aim is to investigate the most common symptoms and predictors or risk factors for pediatric PCC.

**Methods:**

In this umbrella review, the population of interest was defined as children and adolescents from 0 to 19 years old presenting PCC symptoms as defined by the WHO in the International Classification of Diseases. The intervention considered was general follow-up activity to monitor the patients’ recovery status. No comparator was chosen, and the outcomes were symptoms of PCC and predictors or risk factors of developing PCC. Methodological quality, risk of bias and the level of overlap between studies were assessed. A random-effects meta-analytic synthesis of respective estimates with inverse variance study weighting was carried out, for the primary studies included by the reviews retrieved, regarding predictors or risk factors reported.

**Results:**

We identified six eligible systematic reviews, five with meta-analyses, from three databases. The most common symptoms reported were fatigue and respiratory difficulties; female sex and older age were the most reported factors associated with the development of pediatric PCC.

**Conclusions:**

A deeper understanding of pediatric PCC requires well-designed and clearly defined prospective studies, symptom differentiation, and adequate follow-up.

## Introduction

On 5 May 2023, the World Health Organization (WHO) notified the general public of the International Health Regulations (IHR) Emergency Committee’s deliberation regarding the COVID-19 pandemic status. Given the overall decreasing trend in COVID-19-related hospitalizations, deaths and intensive care unit admissions, plus the significantly high level of immunization coverage in the population worldwide, COVID-19 is no longer regarded as a public health emergency of international concern. Although still unknown factors related to the potential development of severe acute respiratory syndrome coronavirus 2 (SARS-CoV-2) are yet to be explored, the recommendation is to move towards implementing strategies for the ongoing management of the COVID-19 pandemic.^1^

Since early 2020, a particular condition following SARS-CoV-2 infection has been detected in many patients, firstly known as the patient-created term ‘long COVID’; in October 2021, the WHO established a clinical case definition of the post COVID-19 condition (PCC) for adults. In children and adolescents, whose health needs were still to be met on the matter, another panel of experts has formulated a separate definition of PCC, in early 2023. Interestingly, as stated for adults, these symptoms may either persist from the initial COVID-19 acute episode or arise after recovery, and fluctuate over time; however, in children and adolescents, diagnosing PCC does not rule out other conditions, potentially overlapping.^2^ The National Institute for Health and Care Excellence (NICE) has instead identified, in 2022, the umbrella term ‘long COVID’ for signs and symptoms that develop or linger after acute COVID-19, which includes two denominations. The first is the ‘Ongoing symptomatic COVID-19’, for ‘signs and symptoms of COVID-19 from 4 weeks up to 12 weeks’ and the second is called ‘Post-COVID-19 syndrome’, for ‘signs and symptoms that develop during or after an infection consistent with COVID-19, continue for more than 12 weeks and are not explained by an alternative diagnosis. […]’. The latter and the WHO’s statement on PCC share one major criterion: overlapping of different groups of symptoms within PCC.

Up to date, several papers have characterized epidemiological aspects,^3^ risk factors^4^ and clinical implications^5^ of adult PCC; nevertheless, its prevalence, most common symptomatology and associated factors are yet particularly unclear in children and adolescents.

In this umbrella review, we aimed to explore the most common symptoms, according to the WHO definition of PCC, and discuss the predictors and risk factors related to the development of PCC in the pediatric population.

## Methods

The aim of an umbrella review is to collect existing syntheses regarding a given topic and provide a summary of their results, addressing a broad research question by combining and comparing findings from systematic reviews, with or without meta-analyses.^6^

The methodologies followed are the one proposed by the Joanna Briggs Institute (JBI) and the Preferred Reporting Items for Systematic reviews and Meta-Analyses (PRISMA) statement.^7^ The Preferred Reporting Items for Overviews of Reviews (PRIOR) checklist was followed as a reporting guideline.^8^ The protocol was prospectively registered in the International Prospective Register of Systematic Reviews (PROSPERO) with the registration number CRD42023396122.

### Inclusion criteria and study selection

The inclusion criteria were outlined in a Population Intervention Comparator Outcome (PICO) model. The population included children and adolescents from age 0 to 19, presenting common symptoms of PCC, as defined by the WHO in the ICD, which is the most recent definition available. The intervention consisted of general follow-up activities to monitor the patients’ recovery status. No comparator was taken into consideration, and the outcomes were defined as symptoms of PCC [fatigue, shortness of breath and cognitive dysfunction (e.g. ‘brain fog’)] and predictors or risk factors (e.g. age, gender, comorbidities, severity of COVID-19 infection) of developing PCC. Only systematic reviews with or without meta-analyses, written in English and published between January 2020 and September 2023 were included; for resource reasons, the research strategy also considered exclusively available full texts. The time inclusion criterion is justified by the fact that the syndrome is exclusively consequent to SARS-CoV-2 infection, which was unknown to the scientific community before the pandemic began. Two authors conducted an independent assessment of the eligibility criteria, assisted by a third one if disagreement were to occur. After eliminating duplicate articles, three authors evaluated titles and abstracts of the articles retrieved, according to the inclusion and exclusion criteria; thereafter, full-text screening of each study was performed by the same researchers. Any disagreement arising during the screening phases was resolved by discussing the criteria with a fourth author.

### Search strategy

PubMed, Scopus and WebOfScience were queried. After specific terms and related relevant keywords were determined and validated in each scientific database, we built a Boolean search string through the combination of Medical Subject Headings (MeSH) terms and unstructured keywords, such as ‘infant*’, ‘adolescent*’, ‘long-COVID’, ‘post-COVID condition’, ‘persistent COVID-19’, ‘post-COVID-19 infection’, ‘long-term effects of COVID-19’, ‘follow-up’, ‘discharge*’, ‘dyspnea’, ‘shortness of breath’, ‘age’, ‘gender’, ‘comorbidit*’. To finalize the search, missing articles were retrieved by hand (i.e. snowball searching).

### Data collection

Two authors, independently and in duplicate, extracted data via a predetermined electronic data collection form. The information retrieved from each study was chosen according to the standardized data extraction tool proposed by JBI and adapted to the requirements for our umbrella review; it included author(s), year of publication and country, number of included studies and relative study designs, age range of the sample, study setting of the review, number of databases queried, PCC predictors/risk factors, PCC symptoms, follow-up time and study results. The datasets generated during and/or analyzed during the current study are available from the corresponding author on reasonable request. Individual patient data were not employed, since aggregated data were sufficient for the study purposes, and due to European Union privacy regulations (GDPR—General Data Protection Regulation—approved with EU Regulation 2016/679 of the European Parliament and Council of the 27 April 2016).

### Data synthesis

The findings extraction and presentation are limited to those presented by the included systematic reviews and meta-analyses. This umbrella review does not include primary research study level data, except for predictors or risk factors, for which we performed a random-effects meta-analytic synthesis of respective estimates with inverse variance study weighting.

Quantitative findings from meta-analyses referring to the nature and prevalence of the most common symptoms, as well as from our meta-analytic synthesis of association measures data, were displayed in a tabular manner, reporting the number of participants from the included studies, the reviews used to determine the outcome(s), and statistical heterogeneity. A ‘Summary of evidence’ table states the intervention and the research synthesis included and provides a succinct and straightforward summary of the results. Qualitative findings for the outcomes of interest were presented in the table format.

### Methodological quality, risk of bias and corrected covered area

The description of methodological quality assessment, risk of bias (RoB) and corrected covered area (CCA) are available in the Supplementary material.

## Results

### Study selection and study characteristics

**Figure 1:**
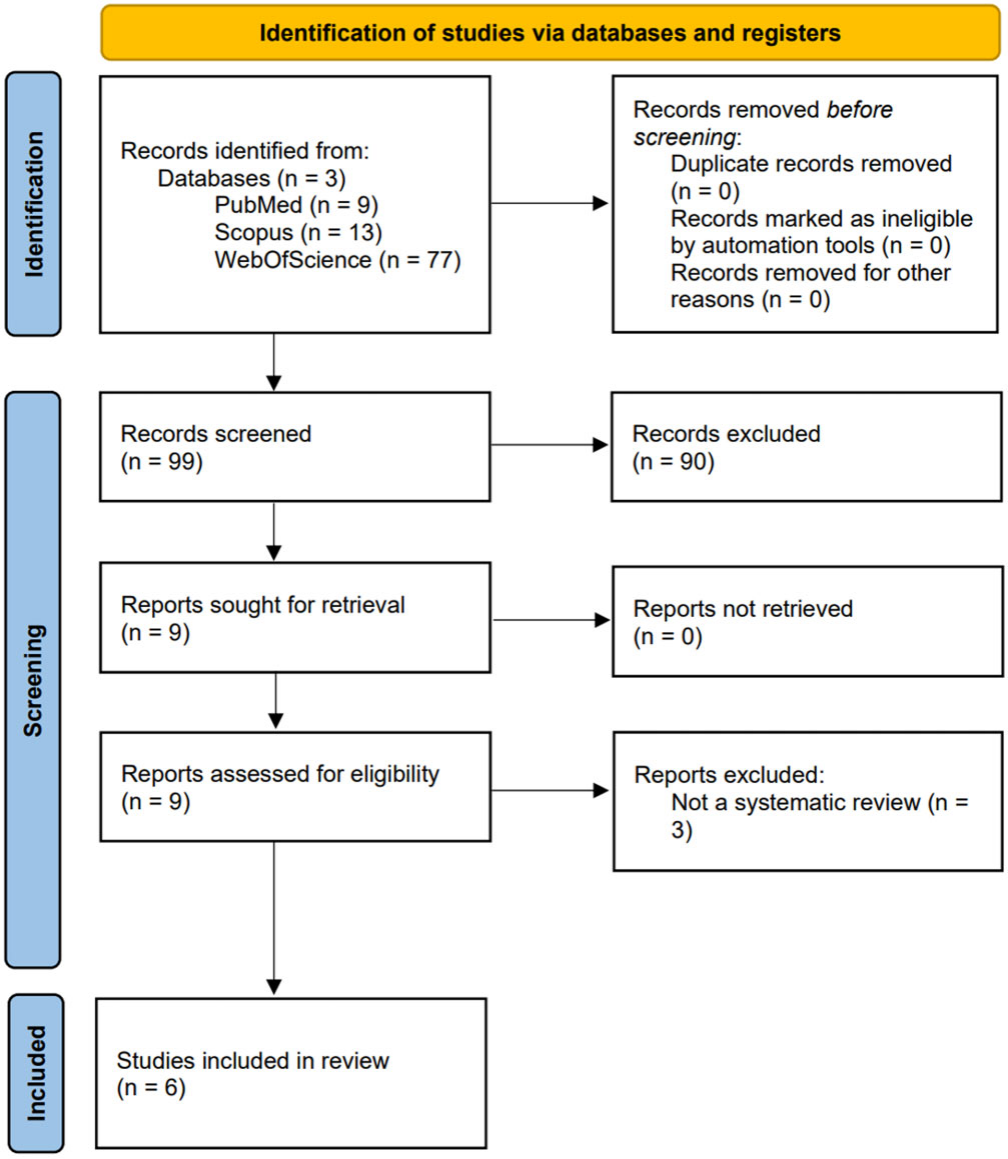
PRISMA flow diagram

Figure 1 depicts the study selection process. The publication period selected to search for the studies was between 2020 and 2023; four^9–12^ of the selected ones were published in 2022, and two^13^^,^^14^ in 2023. Five studies^9–11^^,^^13^^,^^14^ were systematic reviews with meta-analyses, whereas one was a systematic review.^12^ All the included meta-analyses reported the pooled prevalence of PCC in children and adolescents and both the most common symptoms and predictors/risk factors associated, two from age 0 to 18^9^^,^^13^ and three from age 0 to 19.^10^^,^^11^^,^^14^ Sixty-seven percent of studies do not report the study setting, whereas 33%^12^ state ‘inpatients and/or outpatients’ care settings, ‘single center’ and ‘health center’. Only one study^13^ comprised 40 primary studies in their paper; one^14^ included 31 primary studies, two^11^^,^^12^ included 22, one^9^ included 21 and one^10^ included 17. Additional characteristics of the included studies are reported in Supplementary table S1.

### Quality assessment and RoB

The overall quality assessment (Supplementary table S2) and RoB (Supplementary table S3) results for the individual studies are displayed in the Supplementary material.

According to the AMSTAR-2 quality assessment checklist and evaluation, only one study^13^ qualified as ‘high’, since all the domains deemed ‘critical’ by the tool’s authors (Q2, Q4, Q7, Q9, Q11, Q13, Q15) were rated as ‘yes’, and had only one non-critical weakness. Behnood et al. qualified as ‘low’, with one critical flaw and two non-critical weaknesses. The other four studies^9^^,^^10^^,^^12^^,^^14^ qualified as ‘critically low’. Nevertheless, we believe it might be worth noting that the ‘critically low’ rating could be reached by scoring more than one critical flaw, with or without non-critical weaknesses, and the studies appraised showed different degrees of critically low quality. Jiang et al. resulted in ‘no’ for two critical domains (Q2, Q15), but showed no other flaws, critical or non-critical. Pellegrino et al. scored ‘not applicable’ for two critical domains (Q11, Q15) and ‘no’ for other two, along with one non-critical flaw. Lopez-Leon et al. showed five critical flaws and three non-critical; lastly, Campos et al., had six critical and four non-critical flaws.

As far as RoB is concerned, in phase 2, 100% of studies showed low RoB for domain one; 50% of studies^9^^,^^10^^,^^12^ showed high RoB in domain two. For domain three, one^10^ scored high risk, one low risk^14^ and the other four were at unclear risk; 67% of studies^9–12^ was at high risk for domain four. During phase 3, 50% of studies^9^^,^^10^^,^^12^ was deemed at overall high RoB, while the other 50%^11^^,^^13^^,^^14^ was evaluated as at low RoB.

### Corrected covered area

**Figure 2:**
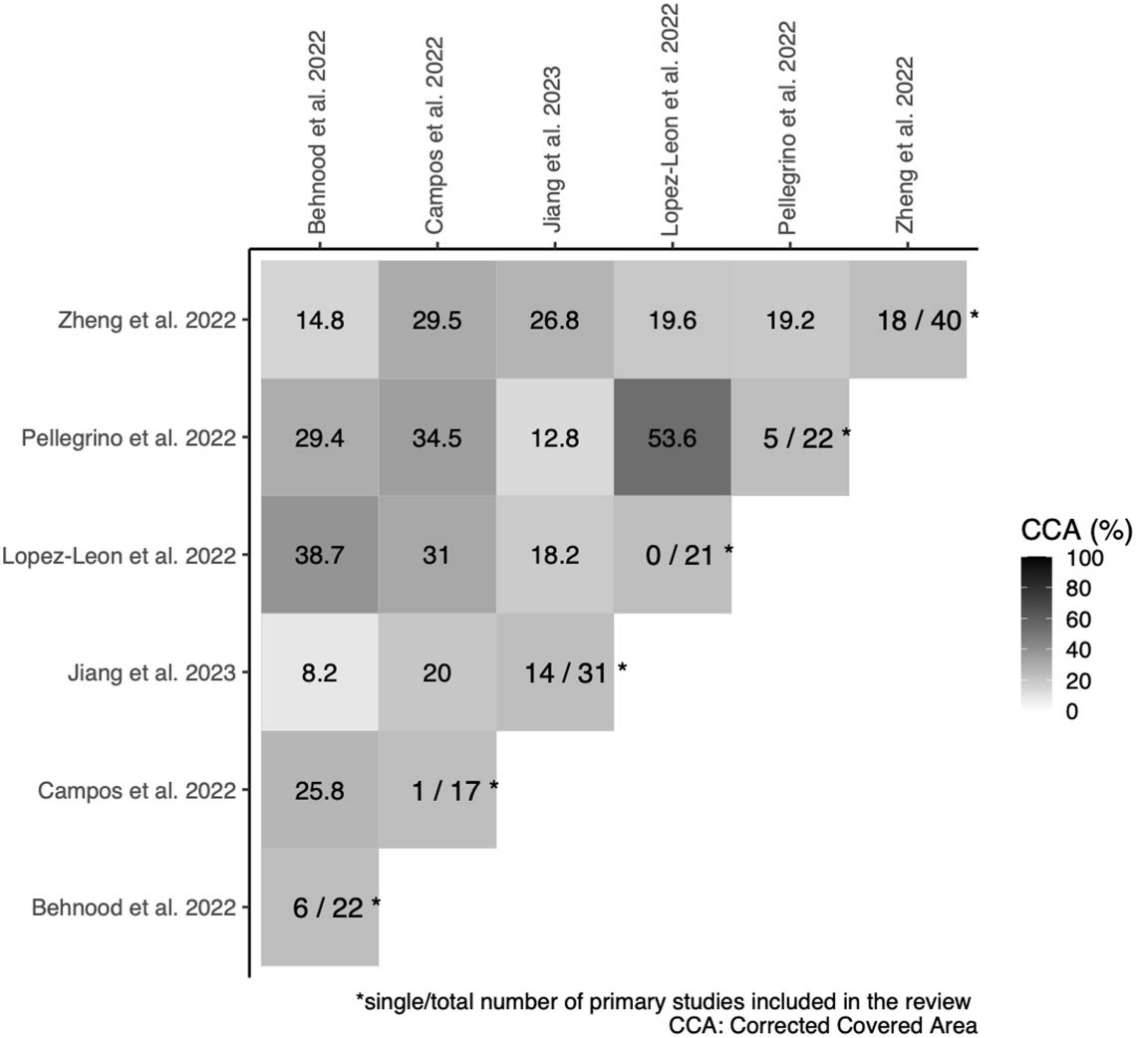
CCA heatmap for paired systematic reviews

The heatmap provided in figure 2 shows the CCA for pairs of systematic reviews (%).

The six reviews included in the present umbrella review summarized the overall evidence from 153 primary studies, including multiple counting (*N*). Despite slight differences in PICO criteria, the overall overlap (CCA) was estimated to be 18.7%, implying a very high overlap. One systematic review^12^ had the highest achievable overlap (i.e. 53.6%) with Lopez-Leon et al. The lowest achieved overlap (i.e. 8.2%), between Jiang et al. and Behnood et al., was classified as moderate.

### Associated factors and most common symptoms

#### Most common symptoms

Current reviews on pediatric PCC list more than 40 different symptoms, whose prevalence varies albeit showing a noticeable pattern of recurrence among the target population. The most common reported were fatigue and respiratory difficulties (i.e. dyspnoea). Only one review^13^ stratified symptoms by mean age, sex ratio, organ system and specific symptom, abnormal imaging and laboratory findings, and severity of COVID-19 disease [presence of multisystem inflammatory syndrome (MIS), severe symptoms during COVID-19 course]. Compared with non-MIS children, MIS patients showed a higher prevalence of PCC in neurologic (29.9% vs. 5.2%; *P* < 0.01), psychiatric (22.6% vs. 2.9%; *P* < 0.01), cardiovascular (9.6% vs. 3.2%; *P* < 0.01) and musculoskeletal (12.2% vs. 1.4%; *P* = 0.04) systems. Moreover, more severely ill children reported a higher prevalence of neurologic (38.3% vs. 10.5%; *P* < 0.01), and psychiatric (42.5% vs. 7.6%; *P* < 0.01) symptoms when compared with non-severe children. Lastly, as far as the sex ratio and percentage of hospitalization at baseline were concerned, there were no statistically significant differences with prevalence of PCC of each organ.

The authors also explored the time progression of the symptomatologic pattern and found a decreasing trend, parallel to the increase in follow-up time: the prevalence of symptoms at three to six months were 26.41% [95% confidence interval (CI) 14.33–40.59; *I*^2^ = 100%], 20.64% at 6–12 months (95% CI 17.06–24.46; *I*^2^ = 31%) and 14.89% (95% CI 6.09–26.51; *I*^2^ = 75%) after more than 12 months. Accordingly, the prevalence of symptoms affecting specific systems showed a significant decrease after 6 months of follow-up: respiratory (*P* < 0.01), psychiatric (*P* < 0.01), neurologic (*P* < 0.01) and cardiovascular (*P* < 0.01) systems. Another review^14^ performed a subgroup analysis of the included prospective cohort studies, based on the duration of follow-up (6, 6–12, ≥12 months), and reported different sets of most common symptoms for each. The highest number of cases (*N* = 5158) had a 3–6 months of follow-up, reporting sore throat, persistent fever, muscle weakness, fatigue and cough.

A detailed list of the most common symptoms reported by each included review is provided in table 1.

**Table 1:** Summary of quantitative findings for the most reported PCC symptoms

Symptom	Author, year	No. of studies/cases^a^	Results (%) 95% CI^b^	Heterogeneity (%)^b^
Fatigue	Lopez-Leon et al., 2022	16/3015	9.66 (4.45–16.46)	99.12
	Campos et al., 2022	14/3182	24 (20–27)	100
	Behnood et al., 2022	15/NA	47(32–62)	NA
	Pellegrino et al., 2022	21/NA	NA	NA
	Zheng et al., 2023	21/1950	20.22 (9.19–34.09)	99.0
	Jiang et al., 2023	8/824	9.4 (4.1–20.2)	97.76
Respiratory symptoms (dyspnea)	Lopez-Leon et al., 2022	9/1387	7.62 (2.08–15.78)	99.15
	Campos et al., 2022	13/1207	16 (14–19)	99
	Behnood et al., 2022	8/NA	43 (18–68)	NA
	Pellegrino et al., 2022	15/NA	NA	NA
	Zheng et al., 2023	11/173	22.75 (9.38–39.54)	94.0
	Jiang et al., 2023	5/386	4.3 (1.1–15.1)	97.08
Headache	Lopez-Leon et al., 2022	13/1875	7.84 (4.04–12.70)	98.49
	Behnood et al., 2022	13/NA	35 (19–51)	NA
	Pellegrino et al., 2022	17/NA	NA	NA
	Zheng et al., 2023	18/1287	15.88 (6.85–27.57)	99.0
	Jiang et al., 2023	7/850	4.6 (1.2–16.2)	97.83
(Arthro)-myalgia	Behnood et al., 2022	10/NA	25 (11–40)	NA
	Pellegrino et al., 2022	15/NA	NA	NA
	Zheng et al., 2023	14/727	11.42 (3.45–22.96)	99.0
Abdominal pain	Behnood et al., 2022	10/NA	25 (9–42)	NA
	Zheng et al., 2023	12/536	12.42 (2.94–26.81)	90.0
	Jiang et al., 2023	4/155	3.7 (2.3–5.8)	53.97
Chest pain/tightness	Campos et al., 2022	11/374	6 (3–8)	100
	Pellegrino et al., 2022	14/NA	NA	NA
Sleep disturbances	Lopez-Leon et al., 2022	8/153	8.42 (3.41–15.20)	93.49
	Jiang et al., 2023	3/70	10.3 (4.9–20.4)	85.99
Cough	Behnood et al., 2022	13/NA	17 (7–27)	NA
	Jiang et al., 2023	8/551	6.8 (2.4–17.7)	97.09
Diarrhoea	Behnood et al., 2022	8/NA	15 (4–26)	NA
	Jiang et al., 2023	2/176	3.5 (1.3–8.9)	89.77
Fever	Behnood et al., 2022	8/NA	18 (5–32)	NA
	Jiang et al., 2023	4/598	10.9 (2.4–38.2)	98.24
Anosmia	Behnood et al., 2022	9/NA	18 (2–34)	NA
Cognitive disturbances	Behnood et al., 2022	10/NA	26 (8–44)	NA
Heart rhythm disturbances/palpitations	Campos et al., 2022	8/344	6 (4–7)	98
Mood disorders	Lopez-Leon et al., 2022	5/730	16.50 (7.37–28.15)	97.49
Reduced activities (functional limitations; decreased exercise capacity)	Campos et al., 2022	2/85; 3/24	48 (25–70); 20 (4–37)	91; 88
Sore throat	Jiang et al., 2023	2/690	14.8 (4.8–37.5)	78.51
Muscle weakness	Jiang et al., 2023	2/17	8.7 (5.5–13.6)	0

a Pellegrino et al. did not report the total number of cases for each primary study included. Behnood et al. reported the total sample size, not stratified in cases and controls, for displaying symptoms’ pooled prevalence.

b Pellegrino et al. did not perform a meta-analysis. Behnood et al. did not report the heterogeneity for symptoms’ pooled prevalence estimates.

#### Most reported predictors or risk factors

Results of the meta-analytic synthesis of predictors or risk factors association measures for developing PCC in children and adolescents are detailed in Supplementary table S4. Twenty studies^15–34^ were cited by the included reviews regarding the presence of predictors or risk factors assessed; however, only 13 of them (63.2%)^15–26^^,^^34^ reported an association measure (in terms of risk ratio or odds ratio) and were thus included in our synthesis. Meta-analytically pooled estimates showed that a significant association with PCC in children and adolescents could be demonstrated for female gender [odds ratio (OR) = 1.72, 95% CI 1.39–2.12], age (OR = 1.09 per 1-year increase, 95% CI 1.05–1.12), severe COVID-19 disease (OR = 2.78, 95% CI 1.78–4.33) and for comorbidities such as obesity/overweight and allergic disease. Moreover, one study^16^ found an association between unvaccinated status and PCC occurrence.

Supplementary table S5 displays qualitative findings, discussed by each included review and accompanied by a comment, concerning the most reported predictors or risk factors. One^9^ did not report any results on the matter, although they provided a brief overview of pediatric predictors or risk factors in the discussion. In the remaining five included reviews, ‘female sex’ was mentioned in all, and ‘older age’ was mentioned in four.^10–13^ ‘Previous long-term conditions/comorbidities (including previous poor mental health)’ were reported in 50% of the included studies,^11–13^ followed by ‘severe COVID-19 disease’ mentioned in 33%^10^^,^^13^ and ‘obesity/overweight’, reported in one.^12^

## Discussion

This umbrella review highlighted that the most common symptoms associated with PCC were fatigue and respiratory difficulties (e.g. dyspnoea). Among the investigated associated factors, female sex and older age were the most reported and likely related to the development of pediatric PCC.

An umbrella review focusing on the same health condition and outcomes, including PCC prevalence, symptoms and risk/protective factors, productivity and socio-economic implications reports quantitative data on adults, but provides no in-depth information on pediatric population.^35^

Despite the ongoing research on risk factors, only one review^10^ addressed the topic of protective factors, particularly vaccination status among children. Indeed, evidence has shown that being unvaccinated would increase the probability of suffering from persistent symptoms 3 months after acute COVID-19 in children.^21^ Another finding that stands out from the results reported earlier is the existence of overlapping PCC symptoms in children and adolescents, interestingly with dysautonomia, underlined by two studies.^9^^,^^10^ Jiang et al. underlined that the varying prevalence of symptoms might be attributable to the broad definition of PCC, which may lead to heterogeneous inclusion criteria and underestimation of point estimates.

Among reported symptoms, heart rhythm disturbances, chest pain/tightness, persistent fatigue, and decreased exercise capacity, are shared with patients suffering from dysautonomia, a dysregulation of the autonomic nervous system that can be triggered by various viruses; similarly to other conditions’ pathogenesis (i.e. chronic fatigue syndrome, fibromyalgia), autoimmune-mediated dysfunction of the autonomic nervous system might contribute to the development of PCC.^36^ Nevertheless, the precise aetiology of dysautonomia in relation to SARS-CoV-2 infection, its interaction with other viruses, or immune-mediated mechanisms, remains uncertain.^9^

These findings raise intriguing implications regarding the multifaceted nature of pediatric PCC.

As for every emerging health condition, exploring potential association and/or causation effects among pediatric patients constitutes the basis for an appropriate development of health policies, which further inform health planning. It is well recognized that health conditions either occurring during developmental years or chronic since birth, could have detrimental and burdensome impacts on the physiological growth and overall well-being of children and adolescents and their families.^37^

Another implication regards the need for training professionals according to the latest and best scientific evidence, both on discerning PCC symptoms and overcoming potential diagnostic hurdles, building multi- and inter-professional teams that include the necessary skilled figures, and sharing knowledge in a network logic to aid effective health improvement globally. In a perspective of healthcare sustainability, children with PCC should be managed not only at the hospital level, but also at the community level to guarantee continuity of care.

Our findings should be assessed in light of its main limitations and strengths. Firstly, an umbrella review cannot be drafted in the absence of previous systematic reviews; the evidence summarized is strictly related to the comprehensiveness, reliability, quantity and overall quality of data reported by the primary studies included in the reviews of interest. However, the methodology chosen is robust to appraise the quality of evidence appropriately and identify potential sources of bias. Furthermore, the existing research on PCC presents several sources of heterogeneity (e.g. lack of stratification regarding demographic data, vaccination status, follow-up times) which hinder the definition of its main symptoms, its impact on public health, and the potential chronicization of the condition. We approached this uncertainty in a systematic and comprehensive manner, focusing on every available study. Although we employed a thorough research strategy and aimed to include all relevant reviews available at the time, it is important to note that this is a rapidly evolving area of study, and our findings offer only a preliminary glimpse of the evidence.

The paucity of extensive evidence on childhood PCC reflects both the novelty of the topic and the complexities that hinder the planning of a scientifically sound primary study (i.e. Randomized Controlled Trial). Further research should prioritize periodic physical and mental assessment, carried out by a multidisciplinary team, of children with PCC. This would generate the necessary primary data to inform both primary studies and tailored health policies, as well as to steer prevention strategies addressing psychophysical wellness during a period as vulnerable as the developmental age is. Furthermore, another priority for future studies should be monitoring the vaccination status of children diagnosed and followed-up with PCC, since emerging evidence highlighted a potential protective effect of vaccines against developing it.^38^

Despite being presented as ‘risk factors’ in the studies, our understanding is that while risk factors, on one hand, are proven to be associated with a health condition, predictors are used to estimate the likelihood of developing a certain disease; regardless, any of these elements have been studied through explanatory and/or prediction models yet, in order to determine their potential causality and/or independent contribution towards developing pediatric PCC.^39^ To date, moreover, there is not a clear association between several and similar aspects of the medical history of children and adolescents with PCC symptoms and its development; hence, we did not consider a univocal naming of these factors (i.e. risk factors) to be appropriate, but rather the proposal of a dual reading, to suggest potential exploration through further research.

Our umbrella review provides an overview of the existing evidence regarding the most common symptoms and associated factors in children and adolescents. Enhancing our understanding of pediatric PCC is of paramount importance, but it requires the implementation of carefully planned prospective studies that incorporate clear and unequivocal definitions of PCC, accurate differentiation of symptoms related to SARS-CoV-2, and sufficient follow-up times. Key priorities should be to plan for tailored health policies to ensure the proper access and continuity of care of this target population, who represent the human capital of the future.

## Supplementary Material

ckae033_Supplementary_Data

## Data Availability

The data underlying this article are available in the article and in its online supplementary material. Key points The most common symptoms reported for pediatric PCC were fatigue and respiratory difficulties; female sex and older age were the most reported predictors/risk factors related to its development. Pediatric PCC symptoms could impair developmental milestones in children and adolescents. Future research should prioritize exploring potential protective factors against developing PCC (e.g. vaccination status).
