# Supplementary material for: Pediatric post COVID-19 condition: an umbrella review of the most common symptoms and associated factors
Source: Eur J Public Health. 2024 Feb 26;34(3):517–23. doi: 10.1093/eurpub/ckae033 (PMC11161168; doi:10.1093/eurpub/ckae033)
Supplement: ckae033_Supplementary_Data [file ckae033_supplementary_data.pdf]

Pediatric Post COVID-19 Condition: an umbrella review of the most common symptoms and associated factors

Supplementary methods

Table S1. Included reviews' characteristics.

Table S2. AMSTAR-2 results.

Table S3. ROBIS results.

Table S4. Meta-analytic synthesis of predictors or risk factors association measures for developing PCC in children and adolescents.

Table S5. Summary of qualitative findings for PCC predictors or risk factors.

Supplementary methods

Methodological quality (AMSTAR-2)

Two authors independently evaluated the methodological quality of the systematic reviews and meta-analyses included by using the AMSTAR-2 checklist (A MeaSurement Tool to Assess Systematic Reviews) (1). It is a quality assessment tool consisting of 16 items, of which seven are deemed to be fundamental: registration of the protocol before starting the review, conducting an adequate and exhaustive literature search, description of grounds for exclusion for individual studies, inclusion of Risk of Bias (RoB) for each study, utilization of appropriate methods for meta-analyses, consideration of RoB when interpreting the review's results, and assessment of publication bias, if any. Quality was categorized based on the number of critical domains met as "critically low" (no or one non-critical weakness), "low" (one critical flaw with or without non-critical weaknesses), "moderate" (more than one non-critical weakness), or "high" (no or one non-critical weakness).

Risk of Bias (ROBIS)

RoB was assessed by two authors independently using the Risk of Bias In Systematic reviews (ROBIS) (2) tool suggested by the JBI (3) framework. The first phase ("assessing relevance") is deemed optional by the ROBIS authors. The second phase identifies concerns with the review process. The third phase allows the judgment of the overall RoB, rated as "low risk", "high risk" or "unclear risk". The results from the assessments, the explanations for the concerns section ratings and the reasoning for each answer were discussed and agreed upon. Furthermore, both for quality assessment and RoB, all the authors have concurred with the choice of tools and cut-offs beforehand; as for weighting the results from AMSTAR-2 and ROBIS, plausible deviations from the methodology were critically discussed and analyzed among all researchers. The choice of using both these tools was shared as well, after exploring the literature regarding the comparison of AMSTAR-2 and ROBIS for umbrella reviews (4).

Corrected Covered Area (CCA)

Additionally, we assessed the Corrected Covered Area (CCA), a method for quantifying numerically, in the form of a percentage, the level of overlap between two reviews. The formula for calculating this index is as follows: $(N - r)/(rc - r)$, where "N" represents the number of publications included in the evidence synthesis,

including multiple counting, "r" is the number of rows, and "c" is the number of columns. The overlap level may be "very high" (CCA >15%), "high" (CCA 11-15%), "moderate", (CCA 6-10%), or "slight" (CCA 0-5%) (5,6); if it occurs, there are four criteria to be followed to choose the review to prefer (most recent publication date, highest AMSTAR-2 score, highest number of studies included or participants, pooled-effect estimates provided or presence of a meta-analysis) (7). Nevertheless, no selection was applied given the paucity of secondary studies meeting the inclusion criteria. Statistical computations were performed by using the statistical software R (version 4.2.2 (8)).

Author(s), country	Title	Included studies' design	N° of databases sourced	PCC symptoms	Follow-up time	Results
Lopez-Leon et al., USA	Long-COVID in children and adolescents: a systematic review and meta-analyses	cross-sectional, cohort (prospective, retrospective)	2 (PubMed, Embase)	mood symptoms (16.50%), fatigue (9.66%), sleep disorders (8.42%), headache (7.84%), and respiratory symptoms (7.62%)	1-13 months	The most common symptoms and percentage of prevalence associated were mood symptoms (e.g., sadness, tension, anger, depression, and anxiety) (16.50%; 95% CI 7.37–28.15, I2 97.49%), fatigue (9.66%; 95% CI 4.45–16.46, I2 99.12%), sleep disorders (e.g., insomnia, hypersomnia, and poor sleep quality) (8.42%; 95% CI 3.41–15.20, I2 93.49%); headache (7.84%; 95% CI 4.04–12.70, I2 98.49%), respiratory symptoms (7.62%; 95% CI 2.08–15.78, I2 99.15%), sputum production or nasal congestion (7.53%; 95% CI 3.78–12.36, I2 0%), cognitive symptoms (e.g., less concentration, learning difficulties, confusion, and memory loss) (6.27%; 95% CI 4.46–8.35, I2 91.32%), loss of appetite (6.07%; 95% CI 3.95–8.59, I2 93.54%), exercise intolerance (5.73%; 95% CI 0.00–19.38, I2 87.77%), and altered smell (e.g., hyposmia, anosmia, hypersomnia, parosmia, and phantom smell) (5.60%; 95% CI 3.13–8.69, I2 97.11%). All other symptoms had less than 5.00% prevalence.
Campos et al., Canada	Long-Term Effect of COVID-19 on Lung Imaging and Function, Cardiorespiratory Symptoms,	cross-sectional, cohort (prospective, retrospective, observational, longitudinal),	3 (PubMed, WOS, Ovid Medline)	[signs] abnormalities in lung imaging abnormal pulmonary function [symptoms]	> 3 months	The pooled prevalence of abnormalities in lung imaging was 10% (95% CI 1–19, I2 = 73%), abnormal pulmonary function was 24% (95% CI 4–43, I2 = 90%), chest pain/tightness was 6% (95% CI 3–8,

	Fatigue, Exercise Capacity, and Functional Capacity in Children and Adolescents: A Systematic Review and Meta-Analysis	case-control		chest pain/tightness, heart rhythm disturbances/palpitations dyspnea/breathing problems fatigue decreased exercise capacity functional limitations		I2 = 100%), heart rhythm disturbances/palpitations was 6% (95% CI 4–7, I2 = 98%), dyspnea/breathing problems was 16% (95% CI 14–19, I2 = 99%), and fatigue was 24% (95% CI 20–27, I2 = 100%). Decreased exercise capacity and functional limitations were found in 20% (95% CI 4–37, I2 = 88%) and 48% (95% CI 25–70, I2 = 91%) of the participants studied, respectively.
Behnood et al., UK	Persistent symptoms following SARS-CoV-2 infection amongst children and young people: A meta-analysis of controlled and uncontrolled studies	cohort (preprint and/or published), cross-sectional (preprint and/or published), case report, matched cohort	3 (Medline, Embase, Cinahl)	cognitive difficulties, headache, fatigue, fever, myalgia, cough, dyspnoea, abdominal, pain, diarrhoea, anosmia/altered sense of smell	median duration 125 days (4.16 months)	Pooled risk difference in post-COVID cases compared to controls (5 studies) were significantly higher for cognitive difficulties (3% (95% CI 1, 4)), headache (5% (1, 8)), loss of smell (8%, (2, 15)), sore throat (2% (1, 2)) and sore eyes (2% (1, 3)) but not abdominal pain, cough, fatigue, myalgia, insomnia, diarrhoea, fever, dizziness or dyspnoea. Pooled prevalence of symptoms in post-COVID participants in 17 studies ranged from 15% (diarrhoea) to 47% (fatigue).
Pellegrino et al., Italy	Prevalence and clinical presentation of long COVID in children: a systematic review	cohort, cross-sectional, case series	2 (PubMed, MedxriV)	fatigue headache arthro-myalgias chest tightness or pain dyspnoea	4-13 months	The most frequently reported symptoms were fatigue (2–87%), headache (3.5–80%), arthro-myalgias (5.4–66%), chest tightness or pain (1.4–51%), and dyspnoea (2–57.1%).
Zheng et al., China	Prevalence and risk factor for long COVID in children and adolescents: A meta-analysis and systematic review	cross-sectional, cohort (prospective, retrospective)	3 (PubMed, Cochrane, Embase)	dyspnea, fatigue headache	0 to 13 months	The generalized symptom (19.57%, [95% CI 9.85–31.52]) was reported most commonly, followed by respiratory (14.76%, [95% CI 7.22–24.27]), neurologic (13.51%, [95% CI 6.52–22.40]), and psychiatric (12.30%, [95% CI 5.38–21.37]).

						Dyspnea (22.75%, [95% CI 9.38–39.54]), fatigue (20.22 %, [95% CI 9.19–34.09]), and headache (15.88%, [95 % CI 6.85–27.57]) were most widely reported specific symptoms. The prevalence of any symptom during 3–6, 6–12, and > 12 months were 26.41% ([95 % CI 14.33–40.59]), 20.64 % ([95 % CI 17.06–24.46]), and 14.89% ([95 % CI 6.09–26.51]), respectively.
Jiang et al., Canada	A Systematic Review of Persistent Clinical Features After SARS-CoV-2 in the Pediatric Population	cross-sectional, cohort (prospective, retrospective	8 (PubMed, Embase, Web of Science, Cochrane Library, WHO COVID-19 Database, China National Knowledge Infrastructure Database, WanFang Database, Latin American and Caribbean Health Sciences Literature)	sore throat, persistent fever, sleep disturbance, fatigue, muscle weakness	3 to 13 months	The 5 most prevalent long-term clinical manifestations after COVID-19 in this pediatric population were sore throat (2 studies, N=3106; pooled estimate 5 14.8%, 95% CI 4.8%–37.5%), persistent fever (4 studies, N= 5128; pooled estimate 5 10.9%, 95% CI 2.4%–38.2%), sleep disturbance (3 studies, N= 697; pooled estimate 10.3%, 95% CI 4.9%–20.4%), fatigue (8 studies, N 5 6110; pooled estimate 5 9.4%, 95% CI 4.1%–20.2%), and muscle weakness (2 studies, N= 196; pooled estimated 5 8.7%, 95% CI 5.5%–13.6%), followed by cough (8 studies, N= 5890; 6.8%, 95% CI 2.4%–17.7%), headache (7 studies, N= 5809; 4.6%, 95% CI 1.2%–16.2%), dyspnea (5 studies, N= 5560; 95% CI 4.3%, 95% CI 1.1%–15.1%), abdominal pain (4 studies, N= 3718; 3.7%, 95% CI 2.3–5.8%)

						and diarrhea (2 studies, N= 3564; 3.5%, 95% CI 1.3%–8.9%). Female gender was found to be associated with a higher risk of developing certain long COVID symptoms, ie, sleep disturbance and headache (P < .01). There were 16.2% (95% CI 8.5%–28.6%) of children and adolescents with a laboratory-confirmed diagnosis of COVID-19 that experienced 1 or more persistent symptom(s) in their latest follow-up, which was ≥3 months post infection (the follow-up duration ranged between 3 to 13 months).

Table S1. Included reviews' characteristics.

	Q1	*Q2*	*Q3*	*Q4*	*Q5*	*Q6*	*Q7*	*Q8*	*Q9*	*Q10*	*Q11*	*Q12*	*Q13*	*Q14*	*Q15*	*Q16*	*OA*
R1	Y	N	N	N	Y	Y	Y	Y	N	N	Y	N	N	Y	N	Y	**CL**
R2	N	N	N	N	Y	Y	Y	Y	N	N	N	N	N	Y	N	Y	**CL**
R3	Y	PY	Y	Y	Y	Y	Y	Y	Y	N	Y	N	Y	Y	N	Y	**L**
R4	Y	N	N	N	Y	Y	Y	Y	Y	Y	NA	NA	Y	NA	NA	Y	**CL**
R5	Y	Y	Y	Y	Y	Y	Y	Y	Y	N	Y	Y	Y	Y	Y	Y	**H**
R6	Y	N	Y	Y	Y	Y	Y	Y	Y	N	Y	Y	Y	Y	N	Y	**CL**

OA=overall· R1: Lopez-Leon et al.; R2: Campos et al.; R3: Behnood et al.; R4: Pellegrino et al.; R5: Zheng et al.; R6: Jiang et al.

Q2, Q4, Q7, Q9, Q11, Q13, Q15 are the critical domains of AMSTAR-2.

Table S2. AMSTAR-2 results.

Review	Phase 2				Phase 3
	**1. Study eligibility criteria**	**2. Identification and selection of studies**	**3. Data collection and study appraisal**	**4. Synthesis and findings**	**Risk of bias in the review**
Lopez-Leon et al.	☺	☹	?	☹	☹
Campos et al.	☺	☹	☹	☹	☹
Behnood et al.	☺	☺	?	☹	☺
Pellegrino et al.	☺	☹	?	☹	☹
Zheng et al.	☺	☺	?	☺	☺
Jiang et al.	☺	☺	☺	?	☺

☺ = low risk; ☹ = high risk; ? = unclear risk

Table S3. ROBIS results.

Predictor or Risk factor	OR	CI 95%	N. studies	Heterogeneity (I^2)
**Female gender**	**1,72**	**[1.39; 2.12]**	**9**	**80,8%**
**Age [years]**	**1,09**	**[1.05; 1.12]**	**8**	**73,9%**
**Severe COVID-19**	**2,78**	**[1.78; 4.33]**	**6**	**52,6%**
Remarkable symptoms/pneumonia during COVID-19	1,37	[0.65; 2.89]	5	95,7%
Prior hospitalisation	1,40	[0.37; 5.25]	3	82,0%
Prior admission to ICU	3,83	[0.17; 85.81]	2	86,9%
Presence of a chronic condition	1,83	[0.92; 3.64]	3	70,5%
**Comorbidities**				
**Excessive weight/obesity**	**1,72**	**[1.09; 2.72]**	**2**	**0,0%**
**Allergic disease**	**1,68**	**[1.12; 2.51]**	**2**	**0,0%**
Neurological disorders	2,09	[0.60; 7.26]	2	68,1%
Gut problems	1,68	[0.97; 2.90]	2	0,0%
Heart disease	1,58	[0.37; 5.60]	1	-
Ethnicity (reference = White)				
Black	1,31	[0.74; 2.30]	2	38,8%
Asian	0,93	[0.79; 1.09]	1	-
Hispanic	1,11	[0.44; 2.77]	1	-
Multisystem inflammatory syndrome in children (MIS-C)	1,90	[0.38; 3.33]	1	-
Positive antibody serostatus	1,89	[0.47; 7.65]	1	-
SARS-CoV-2 variant (post-Delta)	0,64	[0.35; 1.18]	1	-
**Unvaccinated status**	**2,44**	**[1.25; 4.76]**	**1**	**-**
**Number of organ systems involved**	**1,29**	**[1.04; 1.59]**	**1**	**-**
Invasive mechanical ventilation	0,58	[0.25; 1.31]	1	-
Vasopressor-dependent shock	1,81	[0.67; 4.87]	1	-
Muscle pain on admission	6,73	[1.11; 39.68]	1	-
**Index of Multiple Deprivation (reference = High deprivation)**				
Medium-to-high deprivation	1,18	[0.93; 1.49]	1	-
Medium deprivation	1,00	[0.50; 2.00]	1	-
Medium-to-low deprivation	0,90	[0.70; 1.16]	1	-
**Low deprivation**	0,72	[0.56; 0.93]	1	-

Table S4. Meta-analytic synthesis of predictors or risk factors association measures for developing PCC in children and adolescents.

Predictors or risk factors	Author, year	Synthesized findings[a]	Comments
Female sex	Campos et al., 2022	Female sex, […] [was a risk factor] contributing to long COVID in children and adolescents.	The reviews reported information (from some primary studies) that underlined a majority of female cases versus male cases of PCC in children. No further analysis concerning correlation and/or causation was performed. Jiang et al. did not provide any reference to the specific primary studies reporting female sex as a likely risk factor for pediatric PCC.
	Behnood et al., 2022	[…] persistent symptom prevalence was higher in females (OR 1.79 [95% CI, 1.07 to 2.99]).	
	Pellegrino et al., 2022	As concerns sex, in a Danish matched cross-sectional study, female subjects were more prone to show symptoms lasting more than 2 months than males […]	
	Zheng et al., 2023	Also, three studies […] showed that female were more vulnerable for long COVID.	
	Jiang et al., 2023	[…] only gender was presented in 9 studies. Female gender was found to be associated with a higher risk of developing certain long COVID symptoms, i.e., sleep disturbance and headache ($P < .01$).	
Older age	Campos et al., 2022	[…] older age, […] [was a risk factor] contributing to long COVID in children and adolescents.	Behnood et al. reported precise estimates from two primary studies that explored the risk of developing PCC in children, stratified by age groups. The other reviews reported information without providing further analysis concerning correlation and/or causation.
	Behnood et al., 2022	persistent symptoms were more common amongst CYP aged 6–11 (odds ratio 2.74, 95% CI, 1.37 to 5.75) and those 12–18 years (OR 2.68, 95% CI, 1.41 to 5.4) compared to those aged <2 years. Older CYP (12–17 years) were more likely to manifest symptoms ≥28 days in comparison with younger	

		CYP (5–11 years) (5.1% vs. 3.1%).	
	Pellegrino et al., 2022	Older age as a risk factor for persistent symptoms after SARS-CoV-2 infection has been reported in 9 studies.	
	Zheng et al., 2023	[…] pointed out the being older age was associated with higher risk for long COVID.	
Previous long-term conditions/Co morbidities (including previous poor mental health)	Behnood et al., 2022	[persistent symptoms were more common] as well as amongst CYP with a history of allergic diseases (OR 1.67, 95% CI, 1.04 to 2.67).<br><br>[prevalence of persistent symptoms was most reported in] CYP with long-term health conditions (OR 2.95 [95% CI, 1.59 to 5.45]). [another study reported that] having poorer physical and mental health before COVID-19 were important risk factors.	Allergic disease was reported by Behnood et al. with estimates, as well as previous long-term conditions, and by Pellegrino et al. and Zheng et al. without quantitative data. Besides being stated (with or without prevalence data), previous poor mental/physical health conditions were neither further explained nor explored by the reviews. Zheng et al. mentioned neurological conditions as relevant comorbidities for PCC development.
	Pellegrino et al., 2022	[…] allergic disease and previous long-term conditions have been identified as possible risk factors for long COVID […]	
	Zheng et al., 2023	Patients with poor<br><br>physical or mental health were identified impacting long COVID.<br><br>[children were more at risk for PCC if already] affected in specific organs (e.g., allergic diseases, and neurological comorbidities, etc.)	
Severe COVID-19 disease	Campos et al., 2022	[…] multiple symptoms during acute infection, longer hospital stays, and respiratory distress following acute COVID-19 were risk factors	Without further quantitative explanation, two reviews (Campos et al., Zheng et al.) reported that a clinical history of severe COVID-19 disease

		contributing to long COVID in children and adolescents.	course was prevalent in pediatric PCC.
	Zheng et al., 2023	In addition, those with more severe symptoms (e.g., symptomatic during the acute phase, hospitalized 48 h or more), […] and had more symptoms at initial infection, are more likely to develop long COVID.	
Obesity/Overweight	Pellegrino et al., 2022	Among studies included in our review, no statistically significant difference in terms of body mass index (BMI) was found between children reporting persistent symptoms and controls.	Pellegrino et al. commented that overweight/obesity was not found to be associated with pediatric PCC, although they point out that Bloise et al. has found a correlation between high BMI and PCC development in children.

CYP = children and young people.

[a] Synthesized findings are reported verbatim.

Table S5. Summary of qualitative findings for PCC predictors or risk factors.

References

1. Shea BJ, Reeves BC, Wells G, Thuku M, Hamel C, Moran J, et al. AMSTAR 2: a critical appraisal tool for systematic reviews that include randomised or non-randomised studies of healthcare interventions, or both. BMJ. 2017 Sep 21;j4008.

2. Whiting P, Savović J, Higgins JPT, Caldwell DM, Reeves BC, Shea B, et al. ROBIS: A new tool to assess risk of bias in systematic reviews was developed. J Clin Epidemiol. 2016 Jan;69:225–34.

3. Aromataris E, Fernandez R, Godfrey CM, Holly C, Khalil H, Tungpunkom P. Summarizing systematic reviews: methodological development, conduct and reporting of an umbrella review approach. Int J Evid Based Healthc. 2015 Sep;13(3):132–40.

4. Perry R, Whitmarsh A, Leach V, Davies P. A comparison of two assessment tools used in overviews of systematic reviews: ROBIS versus AMSTAR-2. Syst Rev. 2021 Oct 25;10(1):273.

5. Pieper D, Antoine SL, Mathes T, Neugebauer EAM, Eikermann M. Systematic review finds overlapping reviews were not mentioned in every other overview. J Clin Epidemiol. 2014 Apr;67(4):368–75.

6. Bougioukas KI, Diakonidis T, Mavromanoli AC, Haidich A. CCAR : A package for assessing primary study overlap across systematic reviews in overviews. Res Synth Methods. 2023 May;14(3):443–54.

7. Pollock M, Fernandes RM, Newton AS, Scott SD, Hartling L. A decision tool to help researchers make decisions about including systematic reviews in overviews of reviews of healthcare interventions. Syst Rev. 2019 Dec;8(1):29.

8. R Core Team. R: A language and environment for statistical computing. [Internet]. Available from: https://www.r-project.org/
